# Folate-Targeted Transgenic Activity of Dendrimer Functionalized Selenium Nanoparticles In Vitro

**DOI:** 10.3390/ijms21197177

**Published:** 2020-09-29

**Authors:** Nikita Simone Pillay, Aliscia Daniels, Moganavelli Singh

**Affiliations:** Nano-Gene and Drug Delivery Group, Discipline of Biochemistry, University of KwaZulu-Natal, Private Bag X54001, Durban 4000, South Africa; 215022769@stu.ukzn.ac.za (N.S.P.); danielsa@ukzn.ac.za (A.D.)

**Keywords:** cancer, cytotoxicity, folic acid, gene delivery, nanoparticle, PAMAM, pCMV-*Luc*-DNA, selenium, targeting

## Abstract

Current chemotherapeutic drugs, although effective, lack cell-specific targeting, instigate adverse side effects in healthy tissue, exhibit unfavourable bio-circulation and can generate drug-resistant cancers. The synergistic use of nanotechnology and gene therapy, using nanoparticles (NPs) for therapeutic gene delivery to cancer cells is hereby proposed. This includes the benefit of cell-specific targeting and exploitation of receptors overexpressed in specific cancer types. The aim of this study was to formulate dendrimer-functionalized selenium nanoparticles (PAMAM-SeNPs) containing the targeting moiety, folic acid (FA), for delivery of pCMV*-Luc*-DNA (pDNA) in vitro. These NPs and their gene-loaded nanocomplexes were physicochemically and morphologically characterized. Nucleic acid-binding, compaction and pDNA protection were assessed, followed by cell-based in vitro cytotoxicity, transgene expression and apoptotic assays. Nanocomplexes possessed favourable sizes (<150 nm) and ζ-potentials (>25 mV), crucial for cellular interaction, and protected the pDNA from degradation in an in vivo simulation. PAMAM-SeNP nanocomplexes exhibited higher cell viability (>85%) compared to selenium-free nanocomplexes (approximately 75%), confirming the important role of selenium in these nanocomplexes. FA-conjugated PAMAM-SeNPs displayed higher overall transgene expression (HeLa cells) compared to their non-targeting counterparts, suggesting enhanced receptor-mediated cellular uptake. Overall, our results bode well for the use of these nano-delivery vehicles in future in vivo studies.

## 1. Introduction

Cancer-related morbidity is projected to overtake cardiovascular-related diseases as the leading cause of global mortality by 2030 [1]. Thus far, chemotherapeutic treatments have been used with some success; however, the lack of targeted treatment has resulted in cytotoxicity in healthy cells, unfavourable bio-circulation, compromised host immunities, incomplete eradication of malignant cells and the onset of multi-drug resistant cancers [2]. Hence, alternative approaches to chemotherapy to overcome these challenges are required. The combination of nanotechnology and gene therapy, employing a nanoparticle (NP) to deliver a therapeutic gene to cancer cells, has gained interest recently. Nanomedicine is the broad term that encompasses the use of nano-sized material for clinical diagnostics and therapeutic purposes in medicine and has become increasingly promising over recent decades [3,4]. Insight into the capabilities of nanomaterials such as liposomes, metal NPs, quantum dots, carbon nanotubes and polymeric nanostructures have recently increased significantly, as they are small, inexpensive and simple to manufacture and allow for the manipulation of their functionalities [5].

Inorganic NPs such as silica, gold, silver, selenium, iron oxides and carbonaceous NPs, have shown anticancer and antioxidant activity in biological systems with the investigation into their therapeutic potential currently ongoing. The ideal purpose of these NPs is to serve as therapeutic nanocarriers that efficiently encapsulate and deliver therapeutics to an intended target site, with increased bio-circulation, biodegradability and nontoxicity [4]. Selenium has garnered significant attention in the field of nanomedicine due to its promising physicochemical properties, its antioxidant and anticancer activity, its biocompatibility (as an essential micronutrient), its low toxicity at recommended doses, and its reported chemopreventative and therapeutic properties [6,7]. Selenium nanoparticles (SeNPs) have since emerged as a novel form of selenium, carving a niche in nanomedicine as nanocarriers, by virtue of their unique and favourable properties [7]. However, SeNPs are unstable in solution and tend to aggregate rapidly. Furthermore, they cannot bind and protect therapeutic agents as their surface functionality is neutral [8]. The surface functionalities of bare NPs can be manipulated through the addition of biocompatible molecules or polymers [9]. 

Cationic polymers, such as PAMAM (poly(amidoamine) dendrimers (generation 5, G5)), can effectively encapsulate inorganic NPs [10] and have been associated with good transfection efficiency. This polymer is spherical, highly stable and covered in a multitude of positively charged, reactive terminal amine (NH_2_) groups [11]. Cationic dendrimers, in particular PAMAM, due to nonspecific interactions of the primary amine groups with the cell membrane and potential toxicity, pose a concern for their application in gene therapy [12]. Once the inorganic NPs are encapsulated by the cationic polymer, the toxicity of the polymer is reduced, together with aggregation of the NP in solution due to increased stability, allowing the NP to safely bind and compact anionic therapeutic agents (including therapeutic nucleic acids). This can help conserve the integrity of the therapeutic agent during gene delivery until its cytosolic release [12,13]. 

Selective therapy is crucial to prevent the unwanted cytotoxicity seen in conventional chemotherapy. Although NPs can enter tumour cells by the enhanced permeability and retention (EPR) effect, they also possess the added advantage of cell-specific targeting by attachment of a targeting ligand to the NP [14,15]. This allows targeting of receptors that are exclusively overexpressed on specific cancer cell surfaces [16], e.g., the folate receptor (FR) which has been shown to be overexpressed in cancers including ovarian, thyroid, kidney, endometrial, breast and renal cell carcinomas [17]. These FRs bind avidly to folic acid (FA), a dietary supplement which is intrinsically involved in cellular turnover and accommodates the extreme proliferation of cancer cells [18]. It was reported that nanocomplexes containing FA enhanced intracellular delivery of anticancer agents [19,20], mRNA [15] and siRNA [12,21,22] in cells overexpressing the FR. Hence, upon attachment of FA to the NP, uptake of the therapeutic agent into the specific cancer cells can be significantly increased via specific endocytic pathways as opposed to relying on unpredictable passive uptake. 

In the present study, we synthesized SeNPs, dendrimer-functionalized SeNPs (PAMAM-SeNPs) and their corresponding FA conjugates (PAMAM-FA and PAMAM-Se-FA) and investigated their associated cytotoxicity and luciferase gene expression in three cancer cell lines with low to high levels of folate receptors and in a noncancer cell line. Transfection using pCMV*-Luc*-DNA was used as a proof-of-concept study for the future use of these SeNPs in therapeutic gene delivery.

## 2. Results and Discussion

### 2.1. Nanoparticle Synthesis and Characterization

**Figure 1:**
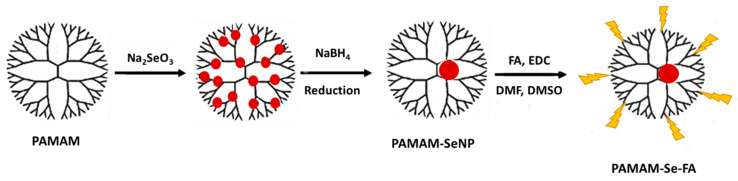
Scheme for the synthesis of poly(amidoamine) dendrimers (PAMAM)-selenium (Se)-folic acid (FA).

Colloidal SeNPs were successfully synthesized using ascorbic acid to reduce the precursor sodium selenite salt. Ascorbic acid is a mild reductant, and the manipulation of its concentration can affect the size and morphology of synthesized SeNPs as well as subsequent antimicrobial and antioxidant activity [23]. The selected concentration of ascorbic acid yielded spherical, favourably sized NPs as seen from electron microscopy and nanoparticle tracking analysis (NTA) results. SeNPs possessed an anionic surface, making them unlikely to bind pDNA or any other negatively charged ligand; hence, surface modification was necessary to reduce aggregation and to improve stability. Figure 1 shows a simple scheme for the production of the final PAMAM-Se-FA.

**Figure 2:**
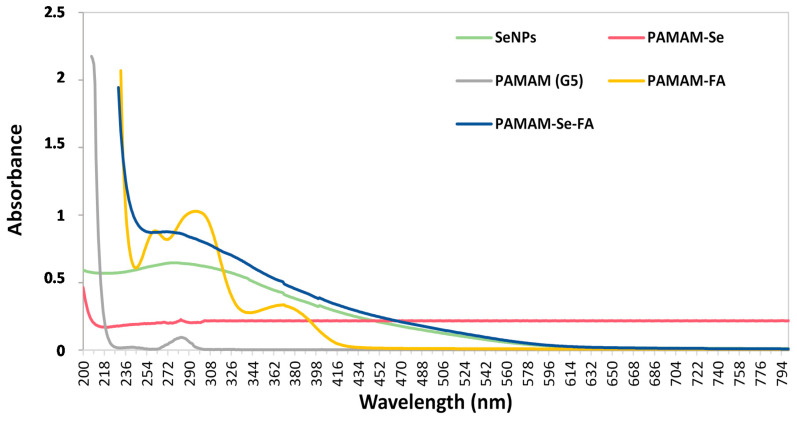
UV-visible absorption spectra of Selenium nanoparticles (SeNPs), PAMAM (G5), PAMAM-SeNPs, PAMAM-FA and PAMAM-Se-FA.

UV-vis spectrophotometry is a simple, analytical method that can confirm the synthesis of a compound in aqueous solution by producing a light absorption spectrum with signatory peaks [24]. UV-vis spectra of the synthesized NPs can be seen in Figure 2.

SeNPs (reduced with ascorbic acid) produced a broad peak centred at 271 nm, indicating that the sodium selenite was chemically reduced into NPs corresponding to results seen in both chemical and green synthesis of SeNPs [15,21,25]. The PAMAM (G5) dendrimer in aqueous solution produced a weak peak at 283 nm, in agreement with reports that this particular peak appears when dendrimers are at physiological pH, and the tertiary amine groups are deprotonated [26]. PAMAM-SeNPs displayed a weak λ_max_ peak at around 282 nm, indicating the presence of the dendrimers. However, the presence of the SeNP bound within the tertiary amine branches may have masked the occurrence of the typical deprotonated polymer peak [27]. The formation of PAMAM-FA (reduced with NaBH_4_) was confirmed by the presence of three broad bands at 261 nm, 296 nm and 371 nm, indicating the covalent attachment of the FA to the NP, as previously reported [28]. The PAMAM-Se-FA (reduced with NaBH_4_) displayed no discernible peak, which may be due to the aliphatic structural nature of the nano-compound [29]. Furthermore, the conjunctive use of the selenium salt and free FA may mask the primary and tertiary amine groups of the dendrimer.

The FTIR analysis successfully confirmed the composition and the presence of SeNPs, PAMAM, PAMAM-SeNPs, PAMAM-FA and PAMAM-Se-FA (Supplementary Figure S1). SeNPs displayed stretch-bonded vibrational frequencies at wave numbers ~2922.16 cm^−1^ and ~2792.41 cm^−1^, indicative of free hydroxyl (O–H) groups. Frequencies at approximately 1081.21 cm^−1^ and 749.26 cm^−1^ corresponded to the C–O–C and C–O groups, respectively. PAMAM (G5) exhibited vibrations at 1181 cm^−1^ and 1453 cm^−1^, denoting amide II, and at 3882 cm^−1^, signifying –CO–NH– bands [30] characteristic of the dendrimer’s aliphatic structure [27]. Peaks at 1384 cm^−1^ and 1427 cm^−1^ correlating to C–H bonds and stretching amide II bands confirm the PAMAM coating of SeNPs. The weak amide A stretch at 3871 cm^−1^ confirmed the presence of free, unmasked primary amine groups on the PAMAM (G5) surface, suggesting that SeNPs might have been adsorbed onto the dendrimer exterior [27]. PAMAM-FA displayed a similar spectrum to that of PAMAM but with evidence of a downshifted stretching amide II at 1441 cm^−1^ and a bending amide at 876 cm^−1^ [31]. The weak amide A stretch at 3904 cm^−1^ confirmed the presence of free primary amine groups of PAMAM. PAMAM-Se-FA displayed a similar spectrum to the PAMAM-FA, with a downshifted anti-symmetrical stretching amide II at 1492 cm^−1^ and a bending amide C–N stretch at 1048 cm^−1^. A weak vibration 3876 cm^−1^ indicated fewer free primary amine groups on PAMAM due to SeNP binding.

Nanoparticle tracking analysis (NTA) provided insight into the size and colloidal stability of the NPs and nanocomplexes, which determines their ability to bind therapeutic and to effectively deliver them to the cells. From Table 1, it can be seen that the size of all the NPs and their nanocomplexes fell into an acceptable range (0–200 nm) that will ensure cellular uptake through either passive or endocytic uptake pathways [32]. It is important to note that NTA size determination is done in an aqueous environment, leading to particles behaving similarly to what may happen during circulation in an in vivo system.

The ζ-potential is representative of the colloidal stability and electrostatic ionic charge at the surface bilayer of an NP. A ζ-potential range of <−25 mV and >+25 mV is considered to indicate stability in solution [33]. PAMAM-containing NPs all depicted stable ζ-potentials, confirming that the PAMAM polymer was highly dispersed and stable in solution due to the dominant, electrostatic repulsive nature of the cationic amine groups on the dendritic surface [34]. PAMAM nanocomplexes without selenium (PAMAM-pDNA and PAMAM-FA-pDNA) reported a decreased ζ-potential (<25 mV), indicating that the inclusivity of SeNPs within the dendrimer reduced the loss of symmetry and masking of the cationic charges by the anionic pDNA. The ζ-potential of the SeNP did not fall within this range and is negatively skewed, indicating that the colloid has a higher risk of aggregation (short-term stability) in solution [35]. As predicted, unmodified SeNPs do not possess the necessary traits to bind or deliver therapeutics in vitro.

**Figure 3:**
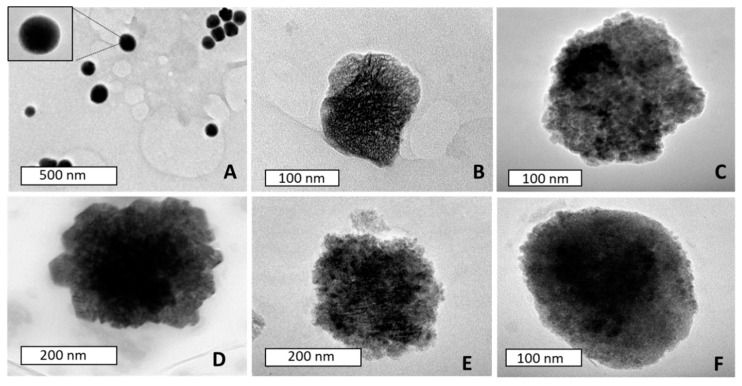
TEM images of (**A**) SeNPs, (**B**) PAMAM-FA, (**C**) PAMAM-Se-FA (**D**) PAMAM-FA-pDNA, (**E**) PAMAM-Se-FA-pDNA and (**F**) PAMAM-Se-pDNA.

Transmission electron microscopy (TEM) images of the NPs and nanocomplexes (Figure 3), displayed monodispersed, spherical SeNPs at sizes similar to that of NTA. These NPs also exhibited a proclivity towards particle aggregation, which is expected, due to the negatively skewed, low ζ-potential seen in NTA. Some reports in the literature have shown PAMAM (G5) dendrimers at much smaller sizes (1–10 nm) [22,27] than that seen in this study (Table 1). TEM results also indicated that these polymers tended to aggregate, a behaviour which could be due to a response to subtle changes in environmental pH. Analysis of the isoelectric points of various dendritic polymers found that dendrimer polymers approaching PAMAM (G5) in size had isoelectric points close to physiological pH, which could cause aggregation [36]. PAMAM-pDNA depicted a smaller size compared to the polymer on its own, which could be attributed to compaction of pDNA on the peripheral surface of the polymer, leading to overall loss of symmetry and size [37]. Although the biomedical field has seen the use of nanomaterials up to the size of 300 nm [38], these NPs and nanocomplexes did not exceed 200 nm, favouring their use. The smaller hydrodynamic size (NTA) could also infer that the dendrimers are more stable in solution and did not aggregate to the extent seen under TEM.

### 2.2. Band Shift Assay

**Figure 4:**
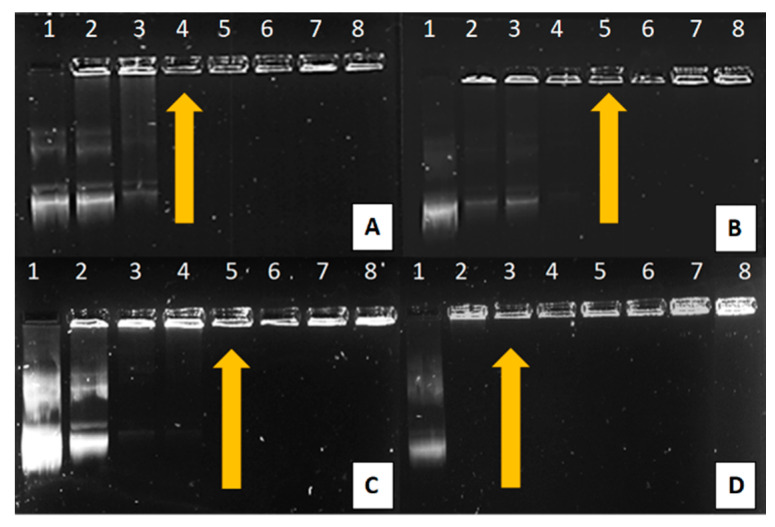
Band shift assay lane 1 (control): 0.25 µg/µL pDNA and lanes 2–8: 0.25 µg/µL pDNA complexed to varying amounts of NP (µg/µL) as follows: (**A**) PAMAM (0.02, 0.04, 0.06, 0.08, 0.10 and 0.12), (**B**) PAMAM-Se (0.25, 0.5, 0.75, 1.0, 1.25 and 1.5), (**C**) PAMAM-FA (0.25, 0.5, 0.75, 1.0, 1.25 and 1.5) and (**D**) PAMAM-Se-FA (0.25, 0.5, 0.75, 0.1, 1.25 and 1.5). Arrows indicate optimal binding of pDNA to NP.

The gel retardation or band shift assay is typically based on determining the affinity between nucleic acids and proteins. In a standard agarose gel separation simulation, negatively charged nucleic acids will migrate towards the positively charged anodic end [39]. This simple procedure can also be used to determine the optimal binding affinity between a nanoparticle vector and a therapeutic nucleic acid, where a cationic particle fully binds to the anionic nucleic acid. This binding prevents the nucleic acid from freely migrating through an agarose gel due to it becoming neutralized. The agarose gel retardation of all synthesized NPs can be seen in Figure 4, with the binding ratios depicted in Table 2.

All NPs depicted efficient binding of the pDNA at relatively low ratios. As PAMAM and the PAMAM-containing NPs increased across lanes 2–8, a greater amount of pDNA became associated with positively charged PAMAM. This was observed as a decrease in the pDNA band intensity that migrated through the gel, accompanied by the pDNA being retained in the wells in the form of nanocomplexes. PAMAM bound the pDNA at the lowest ratio, as at neutral pH, the PAMAM (G5) dendrimers possess ±128 terminal amine groups which are available for ligand binding. As all these cationic groups are unmasked, there is a higher likelihood of fully compacting the pDNA at a lower concentration [27]. The increase in binding ratio for PAMAM-SeNP, PAMAM-FA and PAMAM-Se-FA may be attributed to selenite salt ions and folic acid that may have masked the primary amine groups of the dendrimer, slightly reducing its capacity to bind the pDNA [22,31].

### 2.3. Nuclease Protection Assay

**Figure 5:**
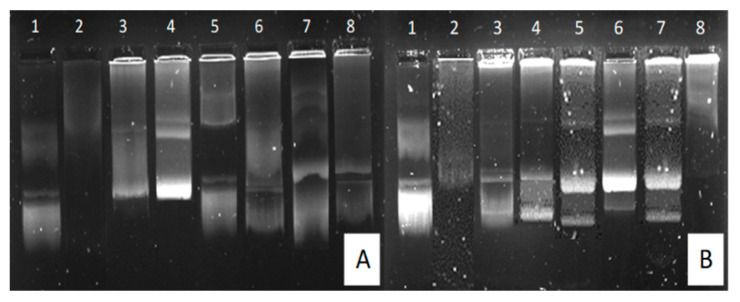
Serum nuclease protection assay depicting pDNA integrity after exposure to serum nucleases: lane 1 (positive control): 0.25 µg pDNA and lanes 2 (negative control): nuclease digested pDNA (0.25 µg). (**A**) Lanes 3–5: PAMAM nanocomplexes and lanes 6–8: PAMAM-Se nanocomplexes. (**B**) Lanes 3–5: PAMAM-FA nanocomplexes and lanes 6–8: PAMAM-Se-FA nanocomplexes.

The nuclease protection assay mimics in vivo conditions to test the NP’s ability to protect the pDNA (through peripheral compaction) and, subsequently, prevent its digestion by serum nucleases. The integrity of the pDNA after exposure to serum nucleases was assessed to determine whether the gene will be viable to express the required protein. The results of the assay can be seen in Figure 5. 

All NPs were able to adequately protect the pDNA at optimal binding ratios (Table 2). PAMAM and PAMAM-Se-FA depicted pDNA degradation at supraoptimal ratios. This degradation may either be indicative of insufficiently protected pDNA or the reduced release of pDNA from the vector due to acute binding, which is more likely as this occurred at the highest tested concentration of the NP.

### 2.4. Ethidium Bromide Displacement Assay

**Figure 6:**
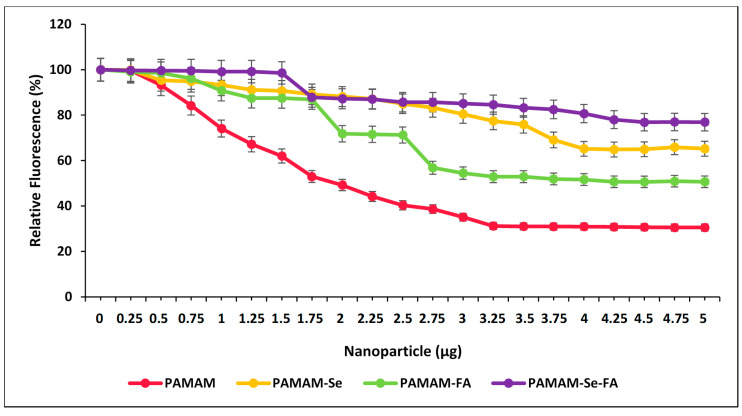
Ethidium bromide displacement depicting the DNA-binding affinity of PAMAM, PAMAM-Se, PAMAM–FA and PAMAM-Se-FA: results are depicted as means ± SD (*n* = 3).

The ethidium bromide (EB) displacement assay is a quantitative assay that shows a decrease in EB fluorescence upon addition of the NP at increasing concentrations. The NP then binds the pDNA and subsequently displaces the EB that has intercalated between the pDNA bases until a plateau in fluorescence is reached. The higher the degree of compaction (more significant fluorescence quenching), the higher the extent of ionic bonding within the nanocomplexes. The results of the assay can be seen in Figure 6.

All the NPs and PAMAM were able to successfully displace pDNA-associated EB, albeit to different extents, depending on their pDNA affinity. PAMAM displayed the highest EB fluorescence quenching of the pDNA, as suggested by the band shift assay. This was expected since all amine groups were available for binding. However, this may prove unfavourable for the release of pDNA in vitro as it may be too tightly bound to the NP. PAMAM-FA and PAMAM-Se-FA both displayed a decrease in the extent of fluorescent quenching compared to the untargeted molecules. This decrease could be attributed to the FA masking PAMAM’s primary amine sites, which can, in turn, impede its association with the pDNA. Hence, more NPs were required to fully compact the pDNA. Interestingly, the presence of the SeNPs seemed to affect the binding efficiency of PAMAM to the pDNA. This may be attributed to the orientation of the cationic charges associated with PAMAM during SeNP surface functionalization, which resulted in a greater number of cationic charges being positioned inwards as opposed to being present on the NP surface.

### 2.5. Cytotoxicity Assay

**Figure 7:**
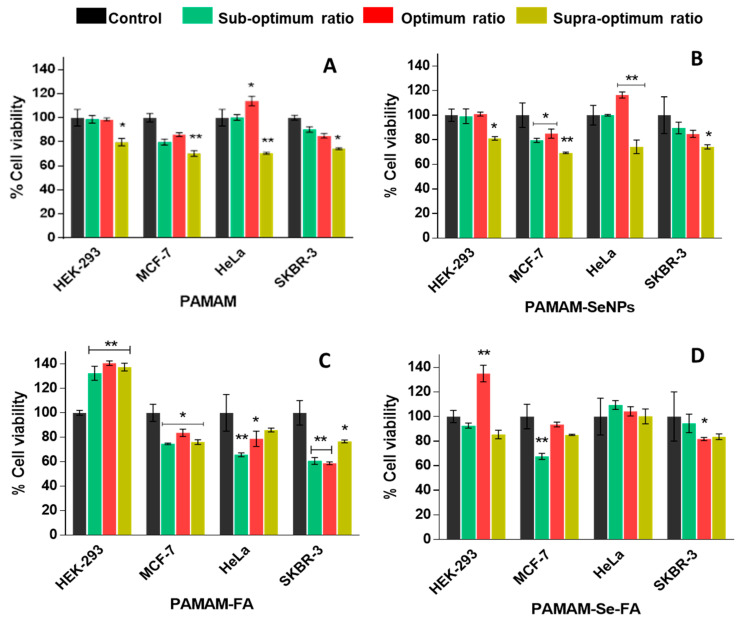
Cell viability (%) of HEK293, MCF-7, HeLa and SKBR-3 cells after treatment with (**A**) PAMAM, (**B**) PAMAM-SeNP, (**C**) PAMAM-FA and (**D**) PAMAM-Se-FA nanocomplexes at the suboptimal, optimal and supraoptimal ratios: the results are depicted as means ± SD (*n* = 3). (* *p* < 0.005 and ** p < 0.01 vs. control).

The 3-(4,5-dimethythiazol2-yl)-2,5-diphenyl tetrazolium bromide (MTT) assay is a colourimetric assay that measures reduction of the yellow MTT salt by mitochondrial succinate dehydrogenases into insoluble, purple formazan crystals [22]. The formazan crystals are then solubilized with an organic solvent (e.g., Dimethylsulfoxide (DMSO)), measured spectrophotometrically and corrected against any background interference. Since the reduction of MTT can only occur in metabolically active cells, the level of activity is a measure of the viability of the cells after exposure to the nanocomplexes [40]. Selenium is an essential micronutrient and is expected to decrease the potential cytotoxic effects of the PAMAM dendrimer on the cell membrane by neutralizing its dominant cationic nature [27]. The addition of FA may also induce a nontoxic growth effect on the cells due to its implicit role in nucleotide synthesis and overall cell turnover [41]. The results of the MTT assay can be seen in Figure 7. PAMAM nanocomplexes displayed a cytotoxic effect at supraoptimal ratios, highlighting the fact that the PAMAM does become cytotoxic at higher doses. This method of cytotoxicity might be attributed to cellular membrane damage, which has been frequently reported in the literature. Naha and colleagues further reported that PAMAM (G_4_–G_6_) dendrimers exhibited a dose- and generation-dependent cytotoxicity in macrophagic mouse cells by forming reactive oxygen species (ROS) that elicited a cascade inflammatory response [42]. PAMAM-SeNP nanocomplexes did not show any appreciable decrease in cell viability which supports reports in the literature stating that the inclusion of SeNPs into dendritic polymers also encourages endocytic entry into the cells, which would reduce overall cytotoxicity [27]. The PAMAM-FA and PAMAM-Se-FA nanocomplexes displayed an increase in cell viability in the control cells, which could indicate that the addition of FA encouraged cell growth since it is an essential vitamin involved in cell turnover [43]. Overall, the observed trends for PAMAM and PAMAM-FA showed higher cytotoxicity in the cancer cell lines than the selenium-containing NPs. This may be a direct result of the increased stabilization and electroneutrality that the selenium provided to the dendrimer, preventing any phospholipid damage in the membrane that would lead to cytotoxicity. As evidenced by their zeta potentials, the Se-containing nanocomplexes had much higher zeta potentials than the PAMAM and PAMAM-FA nanocomplexes, suggesting lower stability of these polymer nanocomplexes, with cationic charges on the PAMAM causing membrane damage even after inclusion of the targeting moiety, folic acid. However, it can be noted that there was some increase in cell viability more so in the normal HEK293 cells than in the cancer cells. The reduction of the MTT salt can change under different culture conditions such as pH or glucose content or based on the physiology of the cells (normal or cancer). Hence, normal cells such as the HEK293 cells may produce more formazan than the cancer cells, leading to higher absorbances being observed [44,45], relating to cell proliferation. Other reasons for increased cell viability could be related to the tested compounds interfering with the enzyme activity or with the MTT salt, leading to an increased absorbances, but since all compounds were tested in all cell lines under the same conditions, it seems that the compounds interacted differently in the various cells possibly due to the reasons given above.

### 2.6. Apoptosis Assay

**Figure 8:**
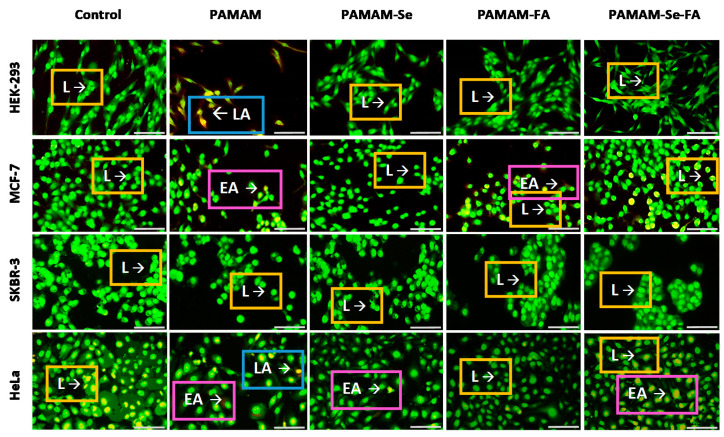
Fluorescent images of dual-stained (acridine orange/ethidium bromide (AO/EB)) cells transfected with PAMAM, PAMAM-SeNP, PAMAM-FA and PAMAM-Se-FA nanocomplexes in HEK293, MCF-7, HeLa and SKBR-3 cells: the stained cells were viewed under an Olympus inverted fluorescence microscope (200× magnification) with a CC12 fluorescence camera (Olympus Co., Tokyo, Japan). Scale bar = 100 µm. L—live cells, EA—early apoptosis and LA—late apoptosis.

**Figure 9:**
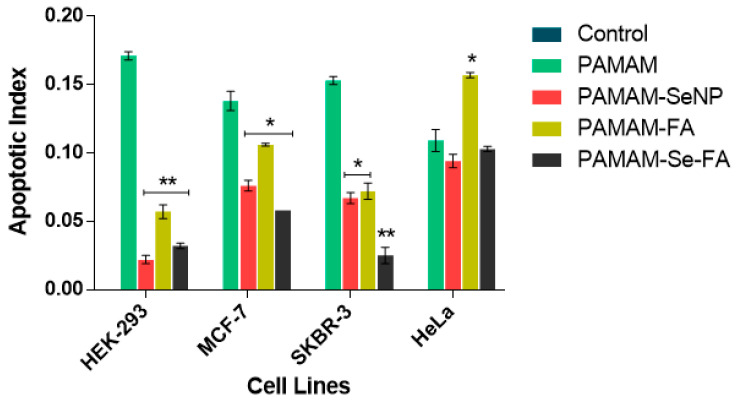
Apoptotic indices calculated for each nanocomplex at the observed optimal ratios in the selected cell lines: the results are depicted as means ± SD (*n* = 3). (* p < 0.005 and ** p < 0.01 vs. PAMAM).

The apoptosis assay was carried out to determine if the potential cytotoxic effects of the nanocomplexes, at observed optimal ratios, were due to their toxicity or as a result of programmed cell death [46]. In this acridine orange/ethidium bromide (AO/EB) dual staining system, AO can perfuse all cells, emitting a bright green fluorescence that is indicative of healthy cell nuclei, while the dominating EB dye pervades cells with compromised cytoplasmic membranes only, leading to emittance of yellow to red fluorescence [47,48]. The fluorescence images (Figure 8) and calculated apoptotic indices (Figure 9) showed that the tested nanocomplexes, particularly the targeting NPs, produced minimal apoptotic activity in the cell lines, indicating that the cytotoxic nature of the dendrimer decreased significantly after functionalization with SeNP or FA. This was determined after multiple comparison significance tests between the Se- and FA-containing nanocomplexes and the PAMAM nanocomplexes alone. These results correlate to the increased cell viability (Figure 7) seen in the selected cell lines after treatment with the Se- and FA-containing nanocomplexes when compared to PAMAM alone, further supporting these results.

### 2.7. Luciferase Reporter Gene Assay and Receptor-Mediated Uptake

**Figure 10:**
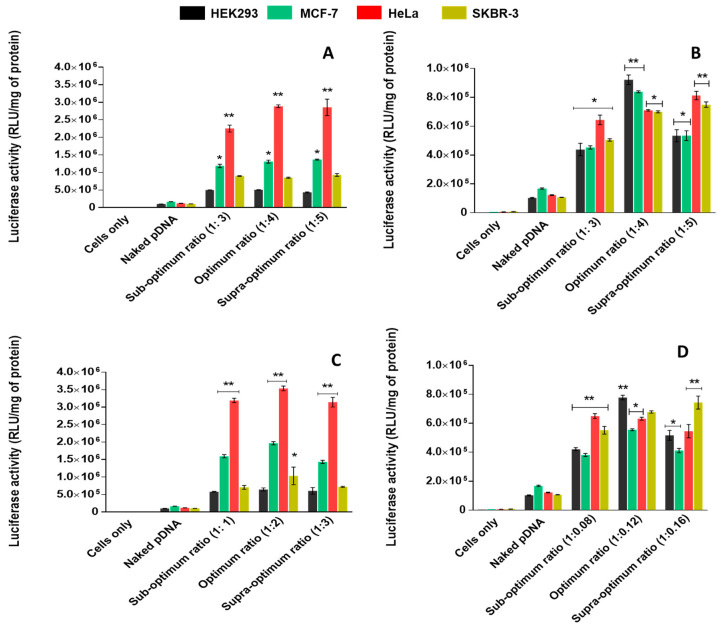
Luciferase activity in HEK293, MCF-7, HeLa and SKBR-3 cells transfected with (**A**) PAMAM, (**B**) PAMAM-Se (**C**) PAMAM-FA and (**D**) PAMAM-Se-FA nanocomplexes at the suboptimal, optimal and supraoptimal ratios: the results are depicted as means ± SD (*n* = 3). (* *p* < 0.005 and ** *p* < 0.01 vs. control).

The plasmid DNA used in this study was the pCMV-*Luc* DNA, which encodes the firefly luciferase gene. Upon successful pDNA delivery in vitro, the gene can be expressed and quantitatively measured by the emitted luminescence. From Figure 10, it was observed that PAMAM-SeNP nanocomplexes displayed higher transgene expression compared to PAMAM nanocomplexes. This may be due to the decreased cationic nature of the PAMAM polymers after inclusion of SeNPs, which ultimately reduced the NP’s unfavourable relationship with the cell membrane and allowed for enhanced cellular uptake [22]. PAMAM-FA and PAMAM-Se-FA depicted their highest gene transfection in the HeLa cells at all tested ratios, with reduced gene expression in the control HEK293 cells. Overall, the targeting NPs displayed much higher transgene expression in the cancer cells compared to their untargeted counterparts. Variation in transfection activity in the different cell lines may be dependent on the cell surface qualities, particularly the quantitative presence of FRs which would regulate the number of nanocomplexes taken up by each cell [31]. Previous studies have shown that HeLa cells express the most FRs compared to MCF-7, which has an appreciable amount of surface FRs and SKBR-3 cells which express the least FRs of the three selected cell lines [43,49,50]. The presence of FA would ideally increase the likelihood of receptor-mediated and caveolae-mediated uptake, whereas untargeted NPs would most likely undergo nonspecific endocytosis [51]. The results also indicate that FR targeting decreased uptake in healthy tissue (HEK293 cells) that minimally expressed these receptors.

Overall, these PAMAM-SeNPs have shown proof of their ability in reporter gene delivery. However, further, in-depth studies are warranted to explore the full potential of these NPs prior to in vivo investigations. Furthermore, the diagnostic and imaging capabilities of these NPs owing to selenium’s photoelectric qualities are yet to be explored in gene therapy. Since these PAMAM-Se-FA nanocomplexes showed significant targeted cellular uptake in cervical cancer (HeLa) cells, they can be exploited further for therapeutic gene delivery or gene silencing in the treatment of cervical cancer.

## 3. Materials and Methods

### 3.1. Materials

Ascorbic acid (C_6_H_8_O_6_, Mw: 176.12 g∙mol^−1^), bicinchoninic acid (BCA) solution, copper (II) sulphate (Cu_2_SO_4,_ MW: 159,609 g∙mol^−1^), dialysis tubing (10 kDa MWCO), dimethylsulfoxide (DMSO), EDC (1-ethyl-3-(3-dimethylaminopropyl) carbodiimide hydrochloride), folic acid, MTT (3-(4,5-dimethyldiazol-2-yl)-2,5-diphenyltetrazolium bromide), PAMAM dendrimer (ethylenediamine core, generation 5.0 (5% w/w in methanol), phosphate-buffered saline (PBS) tablets, N,N-dimethylformamide (DMF), sodium borohydride (NaBH_4_, Mw: 37.83 g∙mol^−1^) and sodium selenite (Na_2_SeO_3_, Mw: 172.94 g∙mol^−1^) were purchased from Sigma Aldrich, St. Louis, MO, USA. Acridine orange hemi (zinc chloride) salt (3,6-Bis (dimethylamino) acridine hydrochloride zinc chloride double salt) (C_17_H_19_N_3_, Mw: 265.36 g∙mol^−1^), 2-[4-(2-hydroxyethyl)-1-piperazinyl] ethane sulphonic acid (HEPES), ethylenediaminetetraacetic acid (EDTA), sodium dodecyl sulphate (SDS) and tris hydroxymethyl-aminomethane hydrochloride (Tris-HCL) were obtained from Merck, Darmstadt, Germany. UltrapureTM agarose was provided by Gibco Invitrogen, Carlsbad, CA, USA. Ethidium bromide and the luciferase assay kit were supplied by Promega, Madison, WI, USA. All other chemicals used were of analytical grade and purchased commercially. Eagles minimum essential medium (EMEM) with L-glutamine, trypsin-versene and penicillin/streptomycin (10,000 U∙mL^−1^ penicillin and 10,000 U∙mL^−1^ streptomycin) were obtained from Lonza Bio Whittaker, Verviers, Belgium. Foetal bovine serum (FBS) was supplied by Hyclone, GE Healthcare, South Logan, UT, USA. The pCMV-*Luc* plasmid DNA was provided by the Plasmid Factory, Bielefeld, Germany. All sterile tissue culture plasticware were obtained from Corning Incorporated (New York, NY, USA). Human embryonic kidney (HEK293), breast adenocarcinoma (MCF-7 and SKBR-3) and cervical carcinoma (HeLa) cells were originally purchased from American Type Culture Collection (ATCC), Manassas, VA, USA. All biological assays were conducted under aseptic conditions in an Airvolution Class II biosafety laminar flow hood. Ultrapure water (18 MOhm) (Millipore, France) was used throughout.

### 3.2. Synthesis of Selenium Nanoparticles (SeNPs)

SeNPs were prepared as described previously [9,15]. Briefly, sodium selenite (5 mL, 0.01 M) was added to ascorbic acid (0.25 mL, 0.04 M) with stirring and reconstituted to a final volume of 20 mL with 18 MOhm water. The solution was left to stir for 2 h at room temperature, followed by dialysis (MWCO 10,000 Da) against 18 MOhm water (1.0 L) for 24 h to remove unreacted material including the reducing agents.

### 3.3. Synthesis of Dendrimer-Encapsulated Selenium Nanoparticles (PAMAM-SeNPs) 

PAMAM-modified SeNPs were prepared using a sodium borohydride reduction of a precursor sodium selenite salt [27]. The PAMAM dendrimer solution was dried by rotary evaporation to remove the methanol. Briefly, 1 mL of PAMAM dendrimer (15 mg∙mL^−1^) in aqueous solution was added to 1.5 mL sodium selenite solution (0.01 M) with stirring. The solution was then left to stir vigorously for 30 min at room temperature. Thereafter, the solution was cooled on ice, followed by the dropwise addition of 1 mL ice-cold NaBH_4_ (0.1 M) to the solution, and was stirred vigorously for 90 min until the solution turned a deep orange-red colour. The solution was centrifuged at 5000 rpm for 20 min, and the pellet was dialyzed as in Section 3.2 to remove the excess reagents.

### 3.4. Synthesis of Folate-Targeted Dendrimer Nanoparticles (PAMAM-FA)

The chemical synthesis for PAMAM-FA was adapted from literature [31], using carbodiimide chemistry to bind the targeting moiety to the NP. FA (9.3 mg) and EDC (56.65 mg) were dissolved and allowed to react with stirring in a mixture containing DMF (6 mL) and DMSO (2 mL) for 1 h. The reaction mixture was then added dropwise to a PAMAM (G5) dendrimer aqueous solution (56 mg∙mL^−1^) and stirred vigorously for 48 h. The resultant solution was dialyzed as in Section 3.2. 

### 3.5. Synthesis of Folate-Targeted Dendrimer-Encapsulated Selenium Nanoparticles (PAMAM-SeNP-FA)

Carbodiimide chemistry was used to conjugate FA to the previously synthesized PAMAM-SeNPs (Section 3.3). FA (1.23 mg) and EDC (5.35 mg) were allowed to react in a mixture containing DMF (3 mL) and DMSO (3 mL) and were stirred vigorously for 1 h. The organic reaction mixture was then added dropwise to a PAMAM-SeNP solution (28 mg∙mL^−1^) and left to stir vigorously for 48 h. The resultant solution was then dialyzed as in Section 3.2.

### 3.6. Preparation of Nanocomplexes

All NP preparations were vortexed (approximately 1 min) and sonicated (approximately 20 min) before use. Varying concentrations of the prepared NPs were added to 0.25 µg of pDNA to obtain the NP:pDNA w/w ratios made up in a constant volume of HEPES buffered saline (HBS) (10 µL, HEPES 20 mM, NaCl 150 mM, pH 7.5). Nanocomplexes were allowed to form at room temperature for 1 h.

### 3.7. UV-Visible and Fourier-Transform Infra-Red Spectroscopy (FTIR)

UV-vis spectroscopy of SeNPs, PAMAM, PAMAM-SeNP, PAMAM-FA and PAMAM-SeNP-FA was carried out in a JASCO-V-730-BIO spectrophotometer (JASCO Corporation, Hachioji, Japan), in a wavelength range 200–800 nm.

FTIR was performed using a Perkin Elmer Spectrum 100 FT-IR spectrophotometer with a universal attenuated total reflectance (ATR) polarization sampling accessory within the wavenumber range 400–4000 cm^−1^ at a 1 cm^−1^ resolution.

### 3.8. Nanoparticle Tracking Analysis (NTA) and Transmission Electron Microscopy (TEM)

Dilutions (1:100, 1 mL) of all NPs and nanocomplexes in 18 MOhm water were prepared. The size, ζ-potential, particle distribution and stability were measured by NTA (Nanosight NS500, Malvern Instruments, Worcestershire, UK). Measurements were conducted at 25 °C at 24 V.

TEM imaging was conducted on a Transmission Electron Microscope (JEOL JEM 1010 at 100 kV (Tokyo, Japan). Approximately 10 µL of all NPs and their corresponding nanocomplexes in suspension were added to copper grids, dried under a UV lamp and analysed using iTEM Soft Imaging System (Tokyo, Japan).

### 3.9. Gel Retardation/Band Shift Assay

Agarose gel electrophoresis was used to determine the optimal binding (w/w) ratios between the NPs and pDNA (0.25 µg). A 1% agarose gel (20 mL) was prepared containing 2 mL of 10× electrophoresis buffer (Tris-HCl 0.36 M, NaH_2_PO_4_ 0.3 M and EDTA 0.1 M pH 7.5) and 4 µL (1 µg∙mL^−1^) EB. Complexes were prepared as previously described, and approximately 3 µL gel loading buffer (sucrose 40% and bromophenol 0.5%) was added before sample loading. The gel was placed in a BioRad Mini-Sub apparatus (Richmond, CA, USA) containing 1× electrophoresis buffer, and electrophoresis was conducted for 90 min at 50 V. Gels were viewed and images were captured using a Vacutec SynGene (Hamburg, Germany) UV-transilluminator gel documentation system.

### 3.10. Nuclease Protection Assay 

Stability of the formed nanocomplexes following serum nuclease-aided digestion was investigated. Nanocomplexes at optimal, suboptimal and supraoptimal (w/w) ratios, obtained from the gel retardation assay, were incubated with 1 µL of FBS at 37 °C for 4 h. EDTA (10 mM) was then added to stop the reaction, followed by the addition of SDS (0.5% w/v) and incubation at 55 °C for 20 min to facilitate the release of the pDNA from the nanocomplexes. The integrity of the pDNA was then assessed using agarose gel electrophoresis as in Section 3.9. Naked pDNA exposed to serum at the same concentration was used as a negative control, and untreated pDNA was used as a positive control.

### 3.11. Ethidium Bromide Displacement Assay

The compactness and strength of NP:pDNA binding was further assessed using the ethidium bromide dye displacement assay. Approximately 0.2 µg of EB in 100 µL HBS was added to a well in a black multi-well plate, and the reading was set as zero fluorescence. Thereafter, 0.5 µg pDNA was added to the mixture and the reading was set as 100% fluorescence. The NPs (1 µL aliquots) were then added, and the fluorescence was measured at excitation and emission wavelengths of 520 nm and 600 nm, respectively, in a GloMax^®^-Multi Detection System (Promega BioSystems, Sunnyvale, CA, USA) until a plateau in fluorescence readings were achieved.

### 3.12. Cytotoxicity Assay

Cells at a seeding density of 2.0 × 10^5^ cells per well were plated into 48-well plates containing 200 µL of growth medium. The cells were incubated overnight at 37 °C to allow the cells to attach, after which the medium was removed and 200 µL fresh medium was added. Nanocomplexes at optimal, suboptimal and supraoptimal (w/w) ratios were added to the wells in triplicate, with untreated cells used as the control (100% viability), and incubated for 48 h at 37 °C. Thereafter, 200 µL medium containing 20 µL of MTT (5 mg/mL in PBS) was added to each well and cells were incubated for 4 h at 37 °C. The medium/MTT mixture was then removed from the wells, and 200 µL of DMSO was added to dissolve the formazan crystals, inducing a purple-coloured dye that was dependent on cell viability and amount of formazan formed. The absorbance was read in a Mindray 96A microplate reader (Vacutec, Hamburg, Germany) at 570 nm, using DMSO as a blank. A reference reading at 630 nm as previously reported was used to compensate for nonspecific signals and was subtracted from the absorbance recorded for the test/treated cells [52].

### 3.13. Apoptosis Assay

Any potential apoptotic induction caused by the nanocomplexes was investigated using the fluorescent acridine orange/ethidium bromide (AO/EB) dual staining assay [47,48]. Cells were seeded into 24 well plates at a cell density of 1.3–1.8 × 10^6^ cells/well and incubated at 37 °C in 5% CO_2_ for 24 h. Thereafter, the medium was removed and replaced with complete medium and nanocomplexes (at optimal ratios) and cells were incubated at 37 °C in 5% CO_2_ for 24 h. The assay was conducted in triplicate, with untreated cells as the positive control. Following incubation, the medium was removed and the cells were washed with 200 µL PBS and stained with 10 µL of AO/EB dye (1:1 v/v of AO (100 mg/mL and EB (100 mg/mL) in PBS) for 5 min. The dye was then removed, and the cells were viewed under an Olympus inverted fluorescence microscope (100× magnification) at an excitation wavelength of 540 nm and an emission wavelength of 580 nm. Images were captured with a CC12 fluorescence camera (Olympus Co., Tokyo, Japan). Apoptotic indices were calculated using Equation (1).

(1) $$\mathrm{Apoptotic}\;\mathrm{Index}\;=\;\frac{\mathrm{Number}\;\mathrm{of}\;\mathrm{Apoptotic}\;\mathrm{cells}\;}{\mathrm{Total}\;\mathrm{number}\;\mathrm{of}\;\mathrm{cells}\;\mathrm{counted}}$$

### 3.14. Luciferase Gene Expression and Receptor-Mediated Uptake 

All cells were seeded and incubated as in Section 3.12. After overnight incubation, fresh medium (including 10% FBS and 1% penicillin/streptomycin) was added to the cells, followed by the addition of nanocomplexes at the optimal, suboptimal and supraoptimal (w/w) ratios. Cells were incubated for 48 h at 37 °C in 5% CO_2_. After that, the medium was removed and the cells were washed with 100 µL PBS. Cell lysis buffer (80 µL) was then added to the cells, which were gently rocked for 15 min. The cells were then scraped from the surface of the plate, and the cell suspensions were centrifuged at 12,000× *g* for 5 s. Approximately 20 µL of the respective cell lysates was added to wells in a 96-well white plate, followed by the injection of 100 µL luciferase assay reagent. Luminescence was measured in a Glomax^®^-Multi+Detection System (Promega Biosystem, Sunnyvale, CA, USA). Protein concentrations of the lysates (50 µL) was determined using a standard BCA assay, and luciferase activity was represented as relative light units (RLUs) per mg protein.

### 3.15. Statistical Analysis

All assays were performed in triplicate (*n* = 3), and quantitative results were conveyed as mean ± SD (standard deviation). Study data were statistically analysed using GraphPad Prism Version 6.0 (GraphPad Software Inc., San Diego, CA, USA). Statistical analysis between means was evaluated using two-way analysis of variance (ANOVA), followed by the Dunnett’s multiple groups mean comparison test. Statistics were performed at 95% confidence interval (CI), and a * *p*-value < 0.05 and ** *p*-value < 0.01 was considered significant.

## 4. Conclusions

The results obtained in this study revealed that these NPs possessed an increased propensity towards effective gene loading, safe intracellular delivery and cellular trafficking and can be deemed as stable, biocompatible choices for the delivery of therapeutics to cancer cells. Evidence has been put forth suggesting the importance and synergistic effect of Se in these NPs, with PAMAM-Se and PAMAM-Se-FA NPs and their nanocomplexes displaying nanoscale dimensions, higher zeta potentials and stability, good cell viability, low apoptotic indices and significant transgenic expression. The supplementation with folate targeting served to increase the efficiency of the targeted nanocomplexes’ in vitro, especially in the folate receptor-rich cells, HeLa > MCF-7 > SKBR-3. PAMAM-Se-FA nanocomplexes were superior in their transfection efficiency. Owing to the versatile nature of these synthesized nano-platforms, they may easily be modified for gene and drug delivery capabilities to potentially treat conditions such as cervical cancer. The synergistic use of bioactive Se in these targeted nanocomplexes can unlock their immense untapped potential in therapeutic gene and drug delivery.

## Figures and Tables

**Table 1:** Hydrodynamic size and ζ-potential for all synthesized nanoparticles (NPs) and their corresponding nanocomplexes.

Nanoparticles	Nanoparticles		Nanocomplexes		
	Size (nm)	ζ-Potential (mV)	pDNA:NP (w/w)	Size (nm)	ζ-Potential (mV)
SeNP	66.6 ± 1.4	−19.4 ± 0.1	-	-	-
PAMAM	125.1 ± 0.3	48.0 ± 6.3	1:0.12	75.2 ± 10.3	21.2 ± 0.6
PAMAM-Se	84.6 ± 13.2	37.1 ± 1.4	1:4	144.0 ± 87.5	28.8 ± 2.8
PAMAM-FA	111.1 ± 6.1	29.9 ± 4.9	1:4	141 ± 33.9	17.1 ± 7.3
PAMAM-Se-FA	135.1 ± 0.4	35.3 ± 0.4	1:2	125.1 ± 10.3	25.9 ± 2.0

**Table 2:** Suboptimal, optimal and supraoptimal ratios of all nanocomplexes.

Nanocomplex	Suboptimal Ratio (w/w)	Optimal Ratio (w/w)	Supraoptimal Ratio (w/w)
PAMAM-pDNA	1:0.08	1:0.12	1:0.16
PAMAM-SeNP-pDNA	1:3	1:4	1:5
PAMAM-FA-pDNA	1:3	1:4	1:5
PAMAM-Se-FA-pDNA	1:1	1:2	1:3

## Supplementary Materials

Supplementary materials can be found at https://www.mdpi.com/1422-0067/21/19/7177/s1.

## Abbreviations

AO	Acridine orange
BCA	Bicinchoninic acid
DMF	N,N-dimethylformamide
DMSO	Dimethylsulfoxide
EB	Ethidium bromide
EDC	1-ethyl-3-(3-dimethylaminopropyl) carbodiimide hydrochloride
EDTA	Ethylenediaminetetraacetic acid
EMEM	Eagles minimum essential medium
EPR	Enhanced permeability and retention
FA	Folic acid
FBS	Foetal bovine serum
FR	Folate receptor
G5	Generation 5
HBS	HEPES buffered saline
MTT	3-(4,5-dimethyldiazol-2-yl)-2,5-diphenyltetrazolium bromide
NPs	Nanoparticles
NTA	Nanoparticle tracking analysis
PAMAM	Poly(amidoamine)
PAMAM-SeNP	Dendrimer functionalized SeNPs
PBS	Phosphate-buffered saline
pDNA	Plasmid DNA (specifically referring to pCMV-*Luc*-DNA in this study)
RLU	Relative light units
SDS	Sodium dodecyl sulphate
SeNPs	Selenium nanoparticles
TEM	Transmission electron microscopy
WC	With competitor
WOC	Without competitor
