# Supplementary figures and images for: Folate-Targeted Transgenic Activity of Dendrimer Functionalized Selenium Nanoparticles In Vitro
Source: Int J Mol Sci. 2020 Sep 29;21(19):7177. doi: 10.3390/ijms21197177 (PMC7584035; doi:10.3390/ijms21197177)

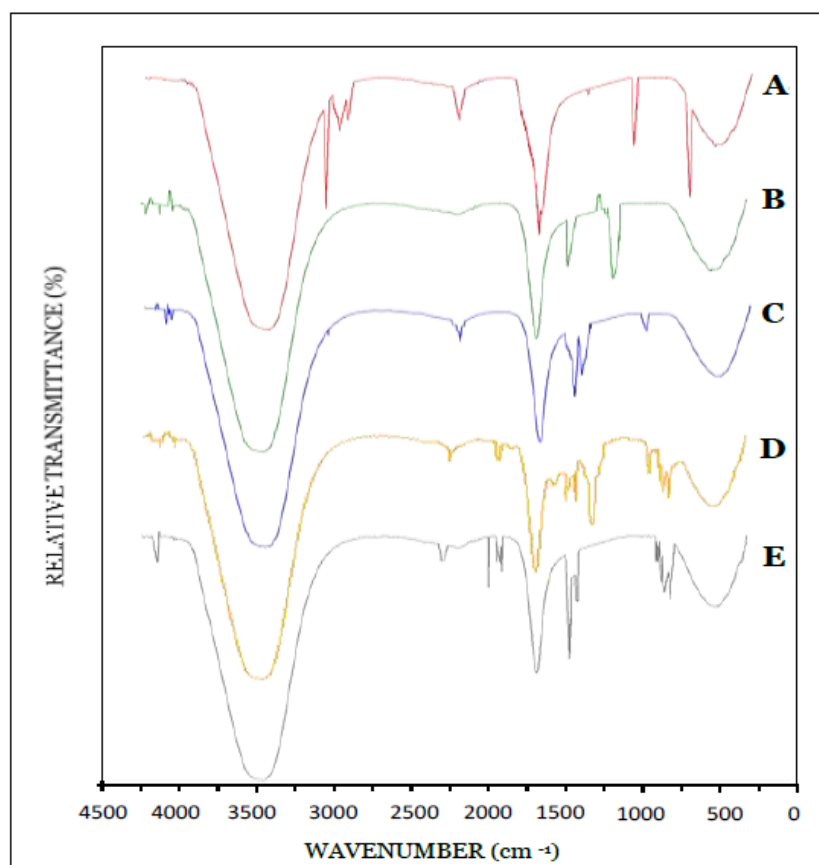

**Figure S1.** FT-IR spectra of (A) SeNPs, (B) PAMAM, (C) PAMAM-SeNPs, (D) PAMAM-FA and (E) PAMAM-Se-FA.

Supplement: Supplementary file 1 [file ijms-21-07177-s001.pdf]
